# Epidemiology of Hepatitis B and C in Republic of Indonesia

**DOI:** 10.5005/jp-journals-l0018-1212

**Published:** 2017-05-05

**Authors:** David H Muljono

**Affiliations:** Eijkman Institute for Molecular Biology, Jakarta, Republic of Indonesia; Faculty of Medicine, Universitas Hasanuddin, Makassar, Republic of Indonesia; Sydney Medical School, University of Sydney, Australia

**Keywords:** Epidemiology, Hepatitis B virus, Hepatitis C virus, Indonesia.

## Abstract

Hepatitis B virus (HBV) and hepatitis C virus (HCV) infections pose serious problems in terms of public health and clinical intervention in a country with approximately 250 million people, who live in more than 17,000 islands. Efforts to combat HBV and HCV have been made through the implementation of universal infant hepatitis B immunization, blood screening, and other health promotion actions, and building epidemiological data to develop intervention strategies. A nationwide study in 2013 revealed hepatitis B surface antigen (HBsAg) prevalence of 7.1%, which indicates that Indonesia has moved from high to moderate endemicity of hepatitis B, leaving the prevalence of 9.4% in 2007. The occurrences of new hepatitis B cases still continue in early childhood period, which may root from low coverage of birth-dose hepatitis B immunization in remote islands, and the potential mother-to-child transmission of HBV from HBsAg-positive pregnant mothers. Other problems still exist including the high HBV infection rates among young adults in remote islands, the presence of occult hepatitis B, as well as the substantial prevalence of HCV infection in general population, who do not have access to diagnosis and treatment. Effective preventive and control strategies are being developed tailored to the local capacity, infrastructures, socioeconomics, and culture, as well as geographical aspects of the country.

**How to cite this article:** Muljono DH. Epidemiology of Hepatitis B and C in Republic of Indonesia. Euroasian J Hepato-Gastroenterol 2017;7(1):55-59.

## INTRODUCTION

Hepatitis B virus (HBV) infection is a major public health problem. Worldwide, approximately 2 billion people have been infected, and more than 240 million are chronic carriers with risk of developing progressive liver diseases, such as cirrhosis, liver failure, and hepatocellular carcinoma (HCC).^1^ The HBV infection accounts for more than 780,000 deaths each year, with HCC currently being the fifth most frequent cancer and the second most common cause of cancer mortality.^2^ The Asia Pacific region has the largest share of HBV and hepatitis C virus (HCV) infection in the world, and 74% of global deaths from liver cancer occur in Asia.^3^ In many countries in this region, there is a lack of robust epidemiological data upon which to develop intervention strategies.

In Indonesia, information about the prevalence of HBV and HCV is lacking for the general population due to several factors, including (1) inadequate disease surveillance systems, with a high likelihood of underreporting of both acute and chronic infections; (2) geographical barriers for successful data collection in a population of about 250 million people distributed in more than 17,000 islands; and (3) limited testing facilities for detection of chronic HBV or HCV, leading to a large proportion of people remaining undiagnosed.^3^

Most studies have been done in different areas or groups of people with risk factors of acquiring this infection, such as blood donors, military members, and indigenous people in isolated areas. Between 1990 and 1997, before the implementation of the national infant universal hepatitis B vaccination, the prevalence rates of hepatitis B surface antigen (HBsAg) among healthy populations in several islands were 4 to 20.3%, categorizing Indonesia as a country with intermediate-to-high endemicity of hepatitis B. The HBsAg prevalence ranged between 37 and 76% in patients with liver cirrhosis, and 37 to 68% in patients with HCC. Data on hepatitis C have also been limited. One among the few data resulted from studies on blood donors in 1998 showed anti-HCV prevalence of 1.5% in Java and 1.0% outside Java.^4^

## ACTIONS TAKEN

A serious effort toward this hepatitis problem started in 1991 with a World Health Organization (WHO)-sponsored universal neonatal vaccination program in Lombok Island.^5^ In 1992, the Indonesian Red Cross (IRC) stepped in to clean up the blood supply; during 1992 to 1994, Indonesia was seen as a model for the international community for clean blood. This effort and recent (2010) active harm reduction measures were considered to support a decreasing incidence.^6^ In 1997, the universal infant hepatitis B program was launched as a national program, and was intensified in the year 1999, when the administration of birthdose vaccination was implemented, started from big islands then gradually expanded to reach smaller islands.

May 2010 was a milestone in the history of fighting viral hepatitis. Indonesia, Brazil, and Columbia were cosponsoring the resolution on hepatitis at the WHO Executive Board session in January 2010, which was adopted by the World Health Assembly (WHA) in May 2010.^7^ This resolution (WHA 63.18) called for a comprehensive prevention of viral hepatitis by all member states, and designated 28 July as the World Hepatitis Day.

Government’s commitment to address hepatitis was made in 2012 by official designation of Hepatitis Control Program within the Indonesian Ministry of Health, secured by the issuance of the Ministerial Decree on the National Control of Viral Hepatitis in 2015.

## CURRENT SITUATION

### National Data of HBV and HCV Infection

Efforts to have national-level data have been made in 2007 through a national surveillance project [Basic Health Survey (Riskesdas)] to collect samples from 21 of 33 existing provinces. The prevalence of HBsAg, anti-hepatitis core antibody (HBc), and anti-HBs was 9.4% (of 10,391 samples), 32.8% (of 18,867 samples), and 30.6 % (of 16,904 samples) respectively. For HCV infection, the prevalence of anti-HCV was 0.82% (of 11,762 samples), with peak incidence in the 50 to 54- and 50 to 55-year-old age groups.^8^ Recently, a nationwide study was conducted through Riskesdas 2013 that covered 33 provinces. Provisional result showed HBsAg, anti-HBc, and anti-HBs prevalence of 7.1% (of 40,791 samples), 31.9% (of 38,312 samples), and 35.6% % (of 39,750 samples) respectively.^9^ It is worthy to note that there has been a decline in the prevalence of HBsAg (9.4% in 2007 to 7.1% in 2013), indicating that Indonesia has moved from high to moderate endemicity of HBV infection.

**Graph 1:**
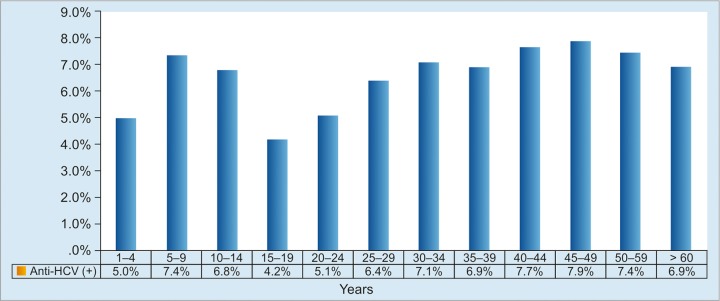
Distribution of HBsAg-positive subjects according to age group. No significant difference is observed between age groups

**Graph 2:**
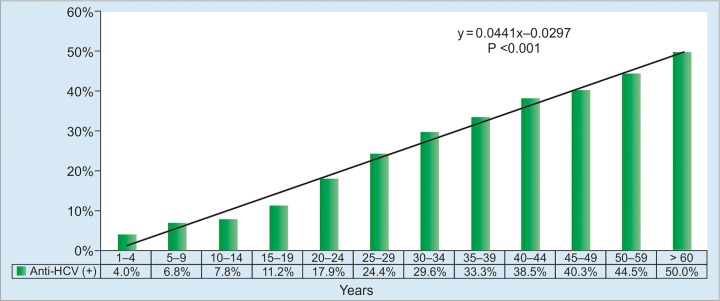
Distribution of anti-HBc-positive subjects according to age group. Linear-by-linear association test shows an increasing trend of anti-HBs-positive rates with increasing age (p < 0.001)

As in other countries, HBV infection has been reduced by the universal infant hepatitis B immunization program; nevertheless, it continues to occur during early childhood period as shown by 5.0% prevalence of HBsAg in the under-5 children (Graph 1). Several reasons could be the background: (1) Uneven coverage of birthdose vaccination, which is lower in eastern part of Indonesia, which consists of small islands separated by sea and are socioeconomically less developed than the islands in the western part; and (2) high HBsAg prevalence in pregnant mothers, which would allow vertical or mother-to-child transmission (MTCT) of HBV infection, particularly in the perinatal period.^10,11^ Anti-HBc prevalence as the evidence of exposure to HBV showed an increasing trend by age, suggesting the high infection rate and role of horizontal HBV transmission in the community (Graph 2).

**Graph 3:**
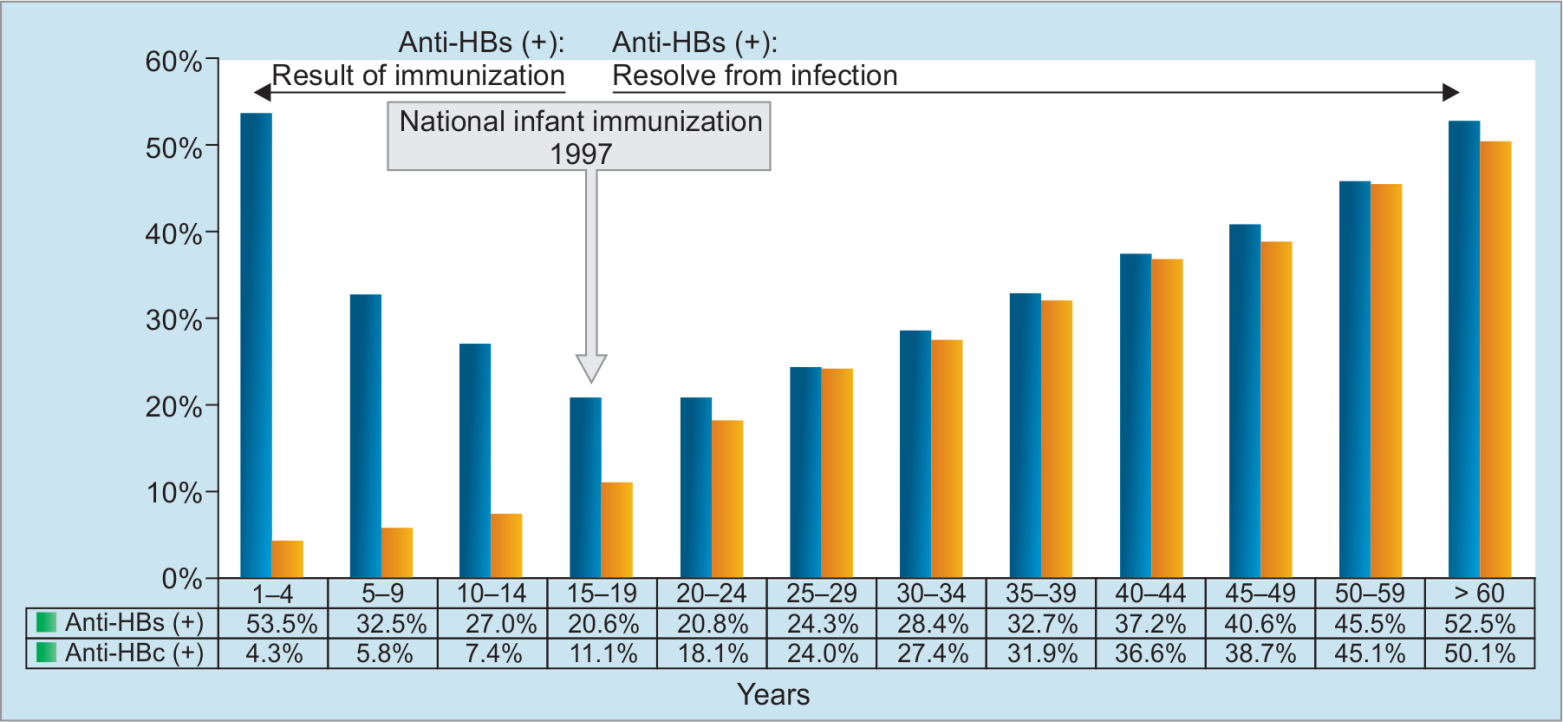
Distribution of anti-HBc-positive subjects and proportion of anti-HBc frequencies according to age. Anti-HBs distribution shows bimodal pattern, highest in 1 to 4 years, lowest in 15 to 19 years, and increased by age in parallel with anti-HBc frequencies

Another interesting finding was the presence of bimodal age distribution of anti-HBs prevalence, which was higher in younger age groups with low proportion of anti-HBc frequencies, decreased to the lowest at 15 to 20 years, and increased in parallel with anti-HBc frequencies (Graph 3). This finding could suggest that anti-HBs positivity in younger age groups was gained by the immunization given to those who were born before 1997 (i.e., the start of national infant immunization program), while in the older age groups, it was obtained through resolved infection. The declined anti-HBs frequency with increasing age could suggest the waning anti-HBs titers by age.

**Graph 4:**
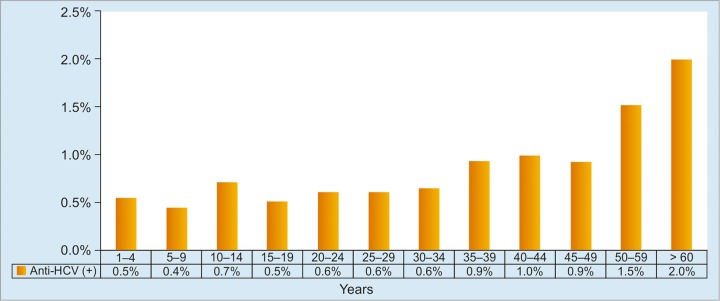
Distribution of anti-HCV-positive subjects according to age group. Anti-HCV rates are highest in the 50 to 59 and >60 year group. No significant difference is observed between age groups

For HCV infection, anti-HCV prevalence was 1.0% (of 40,233 samples) with a peak incidence in subjects aged 60 years and older (Graph 4). The IRC notified between 8,400 and 12,100 individuals of HCV diagnosis annually through blood donation in 2010 to 2014.^12^ Based on IRC and Riskesdas data, it was estimated that there were 1,284,000 (447,000-2,047,000) viremic individuals in 2014. Total viremic infections were estimated to increase slightly to 1,303,000 by 2023 before returning to 1,288,000 by 2030. In 2014, an estimated 9% of the viremic population experienced cirrhosis, HCC, or liver transplant eligibility. By 2030, this proportion was projected to increase to 15%. The number of HCC and decompensated cirrhosis cases was projected to increase through 2030, when cases will number 5,300 and 19,400 respectively, nearly doubling the 2014 values.^6^

### HBV and HCV Infection in Specific Populations

Studies have been conducted among special populations, such as pregnant mothers and injecting drug users (IDUs). One study in 2009 observed HBsAg prevalence of 2.2% among 1,009 Indonesian parturient women in Jakarta, which was markedly reduced compared with the prevalence of 5.2% in 1985. Another study in 2014 revealed HBsAg prevalence of 6.8% (64/943) among pregnant women in Makassar. Of HBsAg-positive subjects, all were HBV deoxyribonucleic acid (DNA) positive, with 15.6% having HBV DNA levels > 6.0 log_10_ IU/mL, which is a recognized as threshold for MTCT.^13^ Other studies reported HBsAg prevalence among pregnant mothers of 4.7% in West Java, 1.9% in Bali, and 3.4% in Mataram.^4,11^ This fact is of concern, because it occurs in pregnant women who tend to be in the immune-tolerant phase of chronic hepatitis B (CHB) with normal physical/laboratory examinations and high-level viremia, but unaware of their HBsAg-positive status and can transmit the virus to their babies.

An ongoing study on 70,000 pregnant women reveals HBsAg prevalence of 2.76%.^6^ With a pregnancy rate of 5,000,000/year, approximately 150,000 pregnant mothers in Indonesia every year have potential to transmit HBV to their babies, of whom 95% may have CHB and become infectious for the entire lifetime. This is of serious concern, as screening tests for HBV in pregnant women are not routinely performed, and antiviral treatment for HBV-infected women has not been adopted as a preventive strategy for MTCT.

Specific studies were also conducted in young adults in Ternate and Banjarmasin representing East Indonesia. Of 376 subjects in Ternate, HBsAg, anti-HBc, anti-HBs, and HBV DNA prevalence was 15.7, 36.2, 24.2, and 27.9% respectively. Of all subjects, 13.0% were HBsAg negative with detectable HBV DNA [occult HBV infection (OBI)], and 56.4% showed negativity for all seromarkers.^14^ Among 195 young adults in Banjarmasin, the prevalence of HBsAg, anti-HBc, and anti-HBs was 4.6, 31.8, and 49.2% respectively, while 37.9% were seronegative for all three parameters, and 6.7% were OBI cases.^15^ These populations showed high hepatitis B prevalence with substantial occurrence of OBI. High percentages of the population were still susceptible and at risk of HBV infection, indicating the necessity to improve preventive strategy including catch-up immunization to susceptible young adults, in addition to the routine infant immunization program.

The HCV infection also appears as another problem. In 2012, 2.5% of the HCV-infected population was active IDU. This percentage was back-calculated using estimates of 70,000 (61,901-88,320) IDU in Indonesia and an IDU HCV prevalence of 77.3% (40-80%), based on data from a recent survey of viral diseases among IDU. Applying a spontaneous clearance rate of 20% suggests there were between 22,400 and 43,680 viremic-infected IDUs.^6,12^

## CONCLUSION

Republic of Indonesia has a substantial burden of HBV and HCV infections. Efforts have been made and supported with increasing commitment by the government. Current data showed that HBV level of endemicity has decreased, entering the WHO category of intermediate endemic region. In general, epidemiological data of HBV and HCV infection are being built, expecting to result in increasing attention to the magnitude of the problem of HBV and HCV infection in more areas of the country. What is clear is that, solutions should engage all sectors to build momentum and work with governments to develop, resource, and implement measures that work toward elimination of viral hepatitis by 2030, as targeted in the Global Health Sector Strategy on Viral Hepatitis 2016 to 2021.^16^ To achieve this goal, there is a need to develop national policies based on up-to-date and reliable epidemiological evidence. Effective preventive and control strategies have to be developed tailored to the local capacity, infrastructures, socioeconomics, and culture, as well as geographical aspects of the country, by the government together with all related stake holders including professional associations, societal participation, with the support of communication media.
